# Toward Standardized pH Measurement for Hardened Cement-Based Materials: Influencing Factors and Measurement Guidelines

**DOI:** 10.1021/acsomega.6c01992

**Published:** 2026-07-21

**Authors:** Payam Shafigh, Poh-Yee Loh

**Affiliations:** † Department of Civil Engineering, College of Architecture and Energy Engineering, Wenzhou University of Technology, Wenzhou 325000, Zhejiang, China; ‡ Centre for Building, Construction & Tropical Architecture (BuCTA), Faculty of Built Environment, Universiti Malaya, Kuala Lumpur 50603, Malaysia

## Abstract

The pH meter is a commonly used instrument in research and industries to test and monitor the pH of a process or material. However, its application in the concrete industry is not well explored. pH measurement for cement-based materials (CBMs) by using a pH meter is challenging because of these materials’ high alkalinity, the fact that they do not exist as aqueous solutions, limited sample size availability, and susceptibility to carbonation. A pH electrode responds to hydrogen (H^+^) ions, but CBMs contain very few H^+^ ions because of their high alkalinity. As a result, accurate pH measurement in these materials requires a high-pH application electrode that is properly maintained to ensure reliable readings, especially for the comparing samples with small pH variation. The aim of this study is to propose guidelines and procedures for pH measurement of hardened CBMs that can be applied universally, reliably, and economically. The variables affecting the sample preparation and pH measurement stages in relation to sample processing, pH meter, and environmental factors are discussed. To accommodate different applications, this study proposes two pH measurement approaches: basic and comprehensive methods. The basic method reports the pH of the solution at the measured temperature and is suitable for the comparison of samples with a pH difference of more than 1. The comprehensive method eliminates the influence of solution temperature and generates a solution temperature-compensated pH (pH_STC_) that represents pH at 25 °C. This method is suitable for the comparison of samples with a pH difference of less than 1 and for pH monitoring across different seasons. Test demonstrations using the developed methods is performed for validation purposes.

## Graphical abstract

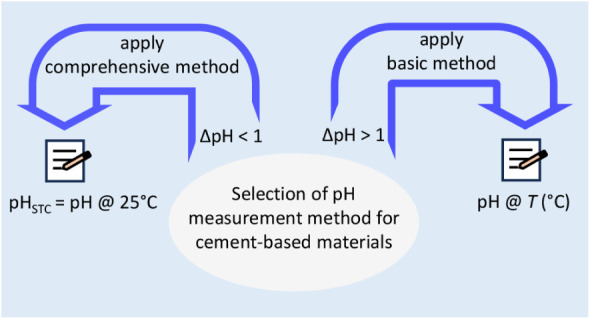

## 1 Introduction

Hydrated Portland cement is highly alkaline because of its portlandite content and the presence of minor constituents of alkali metal oxides such as sodium oxide (Na_2_O) and potassium oxide (K_2_O). Portlandite, chemically known as calcium hydroxide (Ca­(OH)_2_), is formed as a product of cement hydration when water is added to cement. A saturated calcium hydroxide solution provides strong buffering capacity, effectively maintaining pH at approximately 12.45 at 25 °C.^1^ Thus, freshly cast and hardened cement-based materials (CBMs) made from Portland cement theoretically exhibit pH of not less than 12.45 at 25 °C because of the presence of portlandite. Any pH value higher than 12.45 is primarily attributed to dissolved alkali ions (Na^+^ and K^+^) present in the pore solution.^2,3^

For reinforced concrete structures, high-pH CBMs are preferred because they promote the formation of a thin passive film on the embedded steel, protecting it against corrosion. However, carbonation of the cement paste reduces its pH, leading to depassivation of the reinforcing steel, which subsequently triggers steel corrosion and concrete spalling. In deep geological disposal applications, CBMs with a pH less than 11 are favored to prevent chemical deterioration of the bentonite-based backfill that can be caused by the pH of the leachate exceeding 10.^4^ Carbonation, leaching, and pozzolanic reaction induce CBM neutralization, which can be characterized through pH measurement. Teymouri and Atadero^5^ monitored the pH of cement paste at different depths after chloride exposure and performed an accelerated carbonation test. The results showed that 15% fly ash cement paste has a slightly lower pH compared with ordinary Portland cement (OPC) paste (12.95 vs 12.98) after 56 days of curing because of a pozzolanic reaction. After 3 weeks of accelerated carbonation, pH on the surface (0–5 mm) decreases to approximately 11 for OPC paste and 10 for fly ash cement paste. Yu et al.^6^ found that the addition of triethanolamine accelerates the rate of the carbonation reaction of γ-C_2_S under atmospheric pressure by monitoring the pH of the material.

Commonly, the pH of CBMs refers to the pH of their pore solution. The pore solution is the liquid contained within the pores of CBMs that has equilibrated with the surrounding solid phases. The direct way to harvest pore solution from CBMs is through high-pressure extraction with a pore solution extraction device.^7^ However, obtaining sufficient pore solution for pH measurement is challenging, especially for aged (dry) concrete and when testing with a pH electrode. For one-day-old CBMs, only 1–2 mL of pore solution can be extracted from about 250 g of specimens.^3^ Using high pressure of 5,500 MPa, Björk and Eriksson^8^ reported that approximately 30 g of concrete is required to extract 2 mL of pore solution. The maximum pressure used for pore solution extraction in the literature ranges from 200 to 550 MPa for concrete, and from 250 MPa to 1,000 MPa for mortar and paste.^9^ Meanwhile, Constantiner and Diamond^10^ found a slight increase of about 5–8% in OH^–^ ion concentrations in the pore solution of cement paste when the applied pressure increases from 207 to 414 MPa. To address the problem of limited pore solution in hardened samples, Sagüés et al.^11^ misted their samples for 8 weeks, while Pu et al.^12^ used intermittent misting until the samples reached a constant weight before pore solution extraction. Prolonged misting may be attributed to low pH because of the leaching of alkalis^3^ and carbonation, but this phenomenon has not yet been verified.

An alternative approach to the pore solution extraction method is to reproduce the pore solution by leaching CBMs with a known amount of powdered specimen and water until equilibrium is reached. This technique is commonly termed ex situ leaching or cold-water extraction in concrete research. This technique is preferred in recent literature because of its simplicity, speed,^3^ good reproducibility,^8^ and cost-effectiveness.^13^ Moreover, this method is advantageous when the sample size is too small to be processed by using the extraction method.

A pH meter determines the pH of an aqueous solution in accordance with the operational definition established by international organizations such as the International Union of Pure and Applied Chemistry (IUPAC)^14^ and BS1647–1.^15^ In extant literature, aside from direct pH measurement of CBMs by using a pH meter, indirect methods, such as approximating the concentration of OH^–^ ions through titration^16,17^ and calculation from free alkali metal content,^3,18^ were applied. Räsänen and Penttala^17^ and Natkunarajah et al.^18^ found that pH obtained from the titration method is higher than that from direct measurement using a pH meter (0.03–0.45 units and 0.1 unit, respectively). The titration method is suitable for determining the total concentration of a known acid or base. However, it may inaccurately estimate the pH of alkaline solutions containing both alkali metals and alkaline earth metals at varying ratios, such as Portland CBMs. Plusquellec et al.^3^ suggested that the calculation of pH from leachate should involve Na^+^ and K^+^ concentrations only because the concentration for Ca^2+^ will be overestimated because of the dissolution of hydrates. However, these indirect methods were not validated using any standardized reference solution in the studies, and further investigation is required to examine the suitability of using these methods for CBMs. Although the pH meter is a commonly used instrument in research and industries to test and monitor the pH of a process or a material, its accuracy in pH measurement for CBMs is influenced by other factors, which will be discussed in Section 2. Understanding these factors helps minimize errors.

Measuring the pH of CBMs with a pH meter is a more economical approach for studying the neutralization process of CBMs than using other techniques, such as thermogravimetric analysis, X-ray diffraction, and Fourier transform infrared spectroscopy.^2^ However, no structured guidelines have been established for conducting pH measurements in highly alkaline suspensions, such as CBMs. The aim of this study is to develop a guideline and testing method for pH measurement of CBMs that is universally applicable, simple to perform, time-saving, reliable, and cost-effective.

## 2 Variables Affecting pH Measurement

pH measurement of CBMs involves two key stages: (1) preparing the test solution from the solid sample, and (2) measuring pH using a pH meter. Table 1 summarizes the variables associated with these stages; the variables are grouped under the categories of sample processing, pH meter, and environmental. Detailed studies on sample processing factors and temperature effects are available in our previous publications.^19–22^

**Table 1:** Variables Affecting pH Measurement of Cement-Based Materials

	Stage 1 Preparation of test solution			Stage 2 pH determination	
	Matter			Mode	
Variables	Water	Solid	Leachate^a^	Calibration	Measurement
*Sample* *processing*					
Particle size		×			
Water-to-solid ratio	×	×			×
Leaching technique			×		
Leaching time			×		
Filtering method			×		
*pH meter*					
Type of pH meter			×		×
Buffer solutions for calibration				×	
*Environmental*					
Protection of sample from carbonation		×	×		×
Temperature effects		×	×	×	×

a In the form of suspension or filtrate.

### 2.1 Sample Processing

No specific sampling method is available, but as long as the specimen is representative and uncontaminated, any reasonable method can be applied, depending on the purpose of the study. When only a small amount of material can be collected, such as from drilling or scraping the surface of carbonated CBM, the minimum required sample mass depends mainly on the type of pH electrode used, the water-to-solid (w/s) ratio adopted for leaching, and the diameter of the container in which the measurement is performed.

Pulverizing a solid sample into a fine size accelerates the leaching process by increasing the surface area. CBM is sensitive to heat and water, and during powder preparation, precautions should be taken against exposure to heat, exposure to air (to minimize carbonation), and exposure to water (to avoid secondary hydration).^23^ Fine particles, such as those less than 80 μm^24^ or less than 150 μm,^13^ are preferred to accelerate the leaching process. However, processing the entire sample into a very fine particle size requires energy and a long preparation time and may increase the risk of contamination. When the pulverized solid sample is sieved and categorized into different particle sizes, the pH for the large particle size category is lower than that for the small particle size category; however, the pH difference becomes negligible for particle size categories below 600 μm.^17,19,25^ Therefore, processing entire samples into sizes smaller than 600 μm is sufficient for pH measurement via a pH meter.^19^

The purpose of leaching is to obtain equilibrated solutions from the solid CBM. Fresh, unweathered, hardened Portland cement paste is highly alkaline and can saturate test solutions more readily than weathered cement paste. In the extant literature, various leaching techniques, leaching times, and w/s ratios were applied without adequate reasoning. Therefore, a study on these variables was conducted by the authors and published in ref 20. Using a high w/s ratio may result in low pH because of the dilution of ions, whereas a low w/s ratio may reduce ion activity because of excessive concentration of the solution.^26^ Therefore, the w/s ratio should be fixed reasonably. A large volume of well-stirred leachate during pH measurement can reduce sampling bias and improve result reliability. However, when the solid sample is limited, the volume of leachate should meet the requirements of the pH electrode. Currently, most commercial pH electrodes incorporate an indicator electrode (IE), a reference electrode (RE), and a temperature sensor into a single unit (Figure 1). The IE and RE interact with the test solution through the pH-sensitive glass bulb and the reference junction, respectively. Therefore, the volume of the test solution must be sufficient to fully immerse both components during the measurement.

**Figure 1:**
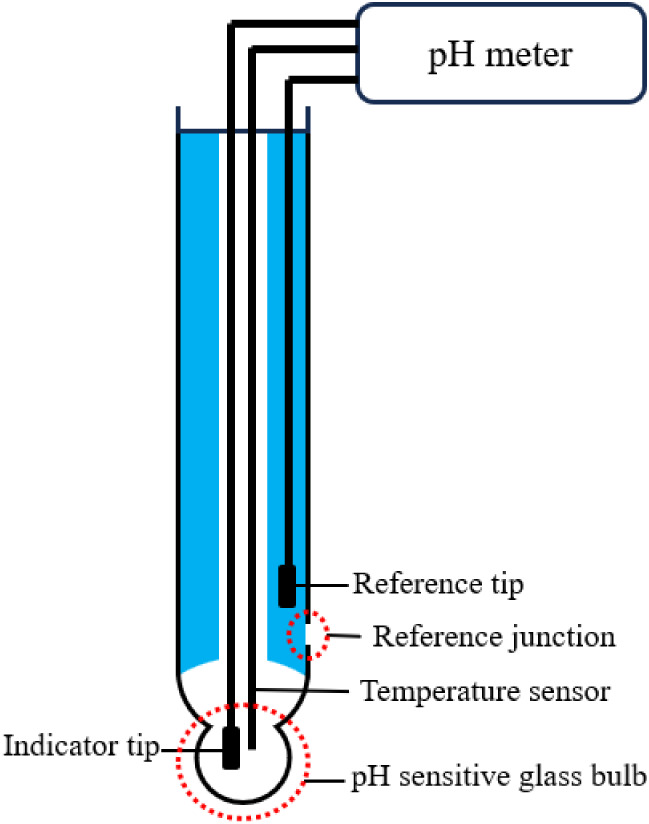
Combined pH electrodes with an indicator electrode (IE, white section) and a reference electrode (RE, blue section).

When measuring pH in a suspension, an unusual additional potential difference called the suspension effect can occur and influence the pH reading.^27^ To minimize this effect, Oman et al.^28^ recommended placing the IE in the sediment and the RE in the equilibrated solution above the sediment, and ASTM D4972–01 ^29^ recommends placing the electrode in the partially settled suspension. To eliminate the suspension effect, Bates et al.^1^ suggested performing pH measurements on the filtrate. Using the filtrate also helps prolong the lifespan of the pH electrode by preventing abrasion of the pH-sensitive bulb and reducing the risk of clogging of the reference junction with suspended solids. Stirring of the sample during pH measurement is also recommended to eliminate concentration gradients and maintain the homogeneity of the solution.^30,31^ However, the influence of stirring on the pH reading of a suspension remains unclear.

### 2.2 pH Meter

The accuracy of pH measurement depends on the type of pH meter and the combination and quality of the buffer solutions used during calibration. The performance of a pH electrode can be observed from its response time and calibration results (e.g., slope and offset readings). Some basic models of pH meters do not provide sufficient information about the calibration results, possibly resulting in a drifting pH reading. Unlike an acidic solution, an alkaline solution is prone to carbonation and may leave salt deposits, such as calcium carbonate, on the pH electrode if not properly cleaned. Therefore, pH electrodes used for measurements in high-alkalinity solutions should be frequently inspected and maintained to ensure optimal performance. Basic maintenance includes topping up or replacing KCl in the electrode when needed, visually inspecting the condition of the reference junction and glass bulb, and soaking the electrode in an acidic solution (e.g., buffer solution with pH 4) for several hours.

Freshly cast CBMs are typically highly alkaline. The pH electrode selected must be specifically designed for high-pH applications to avoid alkaline errors (also known as sodium errors). In highly alkaline solutions, where the sodium ion concentration substantially exceeds the hydrogen ion concentration, sodium ions may penetrate the glass membrane of a pH electrode and be erroneously detected as hydrogen ions.^32^

A pH meter requires frequent calibration using buffer solutions. These buffer solutions are standardized with a known pH–temperature profile, where the naming is based on their pH value at 25 °C. The pH–temperature profile of a buffer solution is usually provided on the product label. A pH meter determines pH by measuring the potential difference between the test and reference solutions. Hence, the reference solution used for calibration should match the test solution as closely as possible to minimize extrapolation errors. A buffer solution with pH higher than 12 or a fresh, saturated calcium hydroxide solution should be prepared as a verification sample of a calibrated test setup for CBM measurement. Given the very low concentrations of H^+^ ions in highly alkaline solutions, the pH-sensitive bulb and the reference junction must be in optimal working condition to produce a reliable result.

### 2.3 Environmental Factors

The two main environmental factors affecting pH measurement of CBMs are carbonation and temperature. When the solid sample is pulverized into small particles with an increased surface area, it become vulnerable to carbonation. Therefore, if pH testing is not performed immediately, solid samples should be stored in a Ziplock plastic bag to avoid contamination from atmospheric CO_2_ and moisture. During leaching, the leachate is stirred to enhance ion dissolution, and gentle stirring is applied during pH measurement to distribute the ions uniformly in the test solution. However, stirring can introduce CO_2_ from the surrounding air, and it may carbonate the leachate and decrease pH. To minimize this effect, we recommend storing the leachate in a cap-seal container and using a small-diameter container during pH measurement. In addition, Sagüés et al.^11^ advised minimizing the exposure of the leachate to the operator’s breathing and surrounding air during handling.

Temperature dependency for pH measurement has two types: temperature dependency of the instrument and temperature dependency of the solution.^33^ pH meters are equipped with an automatic temperature compensation (ATC) feature to mitigate the effect of temperature on electrode performance. However, ATC in a pH meter primarily corrects changes in electrode response (the Nernst slope) rather than accounting for shifts in the thermodynamic equilibrium of the solution.

According to Le Chatelier’s principle, increasing the temperature promotes the dissociation of hydrogen and hydroxyl ions, thereby affecting solution pH.^34^ This temperature effect is highly pronounced in highly alkaline solutions but not as much in acidic solutions.^35^ For example, the pH of a saturated calcium hydroxide solution decreases by 0.033 pH/°C.^1^ In highly alkaline cement pore solutions, temperature variations may further influence chemical equilibria, such as the solubility of calcium hydroxide and ionic activity coefficients. If pH measurement of CBMs is conducted at ambient temperature without intervention on the solution temperature, the measured pH will be higher in winter than in summer, even in the absence of carbonation. Therefore, the pH value and the corresponding measurement temperature should be reported; however, this practice is rarely observed.^33^ Alternatively, we propose reporting the solution temperature-compensated pH (pH_STC_) value when measuring a highly alkaline solution in ref 22. pH_STC_ represents the pH value standardized to 25 °C, and its use provides high measurement accuracy. Although most commercially available pH meters are equipped with ATC features and generate compensated pH values, none can directly generate pH_STC_.

## 3 Guidelines for Test Setup Preparation

The general procedure for pH measurement of hardened CBM involves pulverizing the solid material, sieving it, weighing the required solid and water, leaching in water, filtering the suspension, and measuring the pH of the filtrate, as illustrated in Figure 2. The instruments shown in the figure are merely for reference, and this guideline is prepared on the basis of the literature and outcomes from previous studies on the independent variables, including the published results.

**Figure 2:**
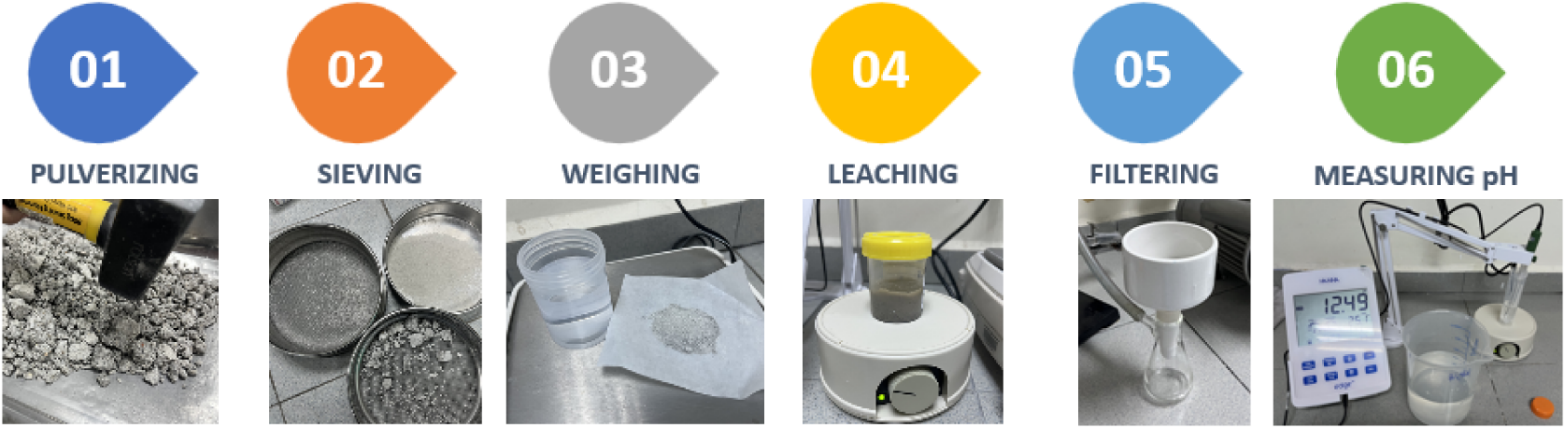
General procedure for pH measurement of cement-based materials. (source: authors).

### 3.1 Pulverizing and Sieving

Our previous study,^19^ which examined the influence of particle sizes and w/s ratios, found that when the entire sample can pass through the No. 30 sieve (600 μm), it is sufficient for leaching using a w/s ratio of 2. A stoning hammer can efficiently break mortar chunks into small particles. However, the crushing should be performed on a tray to contain the fragments and minimize the loss of fine particles. Alternatively, mortar chunks can be ground using a vibratory disc mill at 1,500 rpm for 30 s^3^ or using a pestle and mortar.^24^ However, the latter is difficult to apply to samples containing aggregates.^24^ Notably, excessive pulverization may produce overly fine particles that tend to agglomerate. Fine particles produced by manual crushing, milling, or drilling should be stored in a Ziplock plastic bag to prevent carbonation and contamination. The prepared specimen must accurately represent the composition of the original material. Moreover, the equipment used, such as chisels, hammers, mortars and pestles, drill bits, sieves, ball mill jars, and grinding balls, should be thoroughly cleaned before use to minimize cross-contamination from previous samples. Sieves can be cleaned through dry brushing or by using compressed air.

### 3.2 Weighing

When determining the mass of a solid sample and the water required for pH measurements on CBM, the availability of the solid sample and the type of pH electrode used should be considered. Distilled or deionized water is used for leaching. The volume of water required depends on the mass of the solid sample and the type of pH electrode used. A w/s ratio of 2 is recommended.^20,36^ At a small, low w/s ratio of 1, the high ion concentration reduces ion activity and produces a small pH value when measured with a pH electrode; at a high w/s ratio of 10, the pH of the solution decreases because of water dilution.^20^ When only a limited amount of the solid sample is available (e.g., a carbonated layer that is within a few millimeters from the concrete surface), the volume of the test solution should be planned in accordance with the minimum immersion requirements of the pH electrode. The electrode used must be designed for high-pH applications to minimize alkaline error. For reliable sampling, using less than 5 g of a solid sample per pH test is not advisible,^20^ unless a pH electrode specifically designed for a small amount of solution and high-pH application is employed.

### 3.3 Leaching

During leaching, applying at least 1 min of continuous stirring followed by 10 min of leaching without stirring is sufficient for particle sizes of not more than 600 μm.^20^ Stirring can be performed with a magnetic stirrer and a stirrer bar or via manual hand shake. The suspension should be mixed in a screw-cap container to prevent spillage and carbonation during the leaching process. The stirrer bar should be slightly smaller than the diameter of the container but not smaller than half of the diameter of the container for efficient stirring. The stirring speed of the magnetic stirrer should be adjusted so that the stirrer bar can rotate freely for efficient mixing.

### 3.4 Filtering

The influence of different filteration methods with varying particle sizes on pH measurement of cement mortars was reported.^21^ pH measurement on a filtrate is preferred over suspension because it can eliminate the suspension effect and minimize physical damage to the pH electrode by solid particles. Syringe or vacuum filtering using a Büchner funnel is recommended. Filter media, such as quantitative filter, glass microfiber filter, and nylon membrane, with different pore sizes, have been tested with a pH difference of less than 0.10 unit.^21^ Syringe filtering is suitable for low-volume suspension because it is easy to manage and efficient. Thirty seconds before filtering the suspension, the suspension (in the sealed bottle) is shaken by hand 2–5 times, allowing for sedimentation before filtration then performing pH measurement.

### 3.5 Measuring pH

The pH electrode used should be suitable for high-pH applications. The pH of CBMs normally ranges from 8–13.^37,38^ For measurements within this range, three-point calibration using buffer solutions with pH 7, 10, and above 12 is recommended. For test samples with high pH of not less than 10, two-point calibration using buffer solutions with pH 10 and above 12 is sufficient because the measured pH is determined by the interpolation of the calibration results.^31,32^ Ideally, calibration and measurement should be conducted with the solution temperature maintained at 25 °C. Glass beakers should be avoided to minimize the electromagnetic interference that can arises when a glass pH electrode is used, and a plastic container is preferred for storing the test solution.^39^

## 4 Proposed pH Measurement Methods for CBMs

This study seeks to clarify the misconceptions about pH measurements for CBMs and increase the reliability of the test results. To cater to different scenarios of pH measurement, we propose two methods: a basic method that reports pH together with the solution temperature (Figure 3) and a comprehensive method that reports pH_STC_ (Figure 4). The first method is suitable for comparing samples with a wide pH range, such as carbonated and uncarbonated CBMs. The second method is suitable for strongly alkaline samples (e.g., the influence of admixtures on the pH of CBMs).

**Figure 3:**
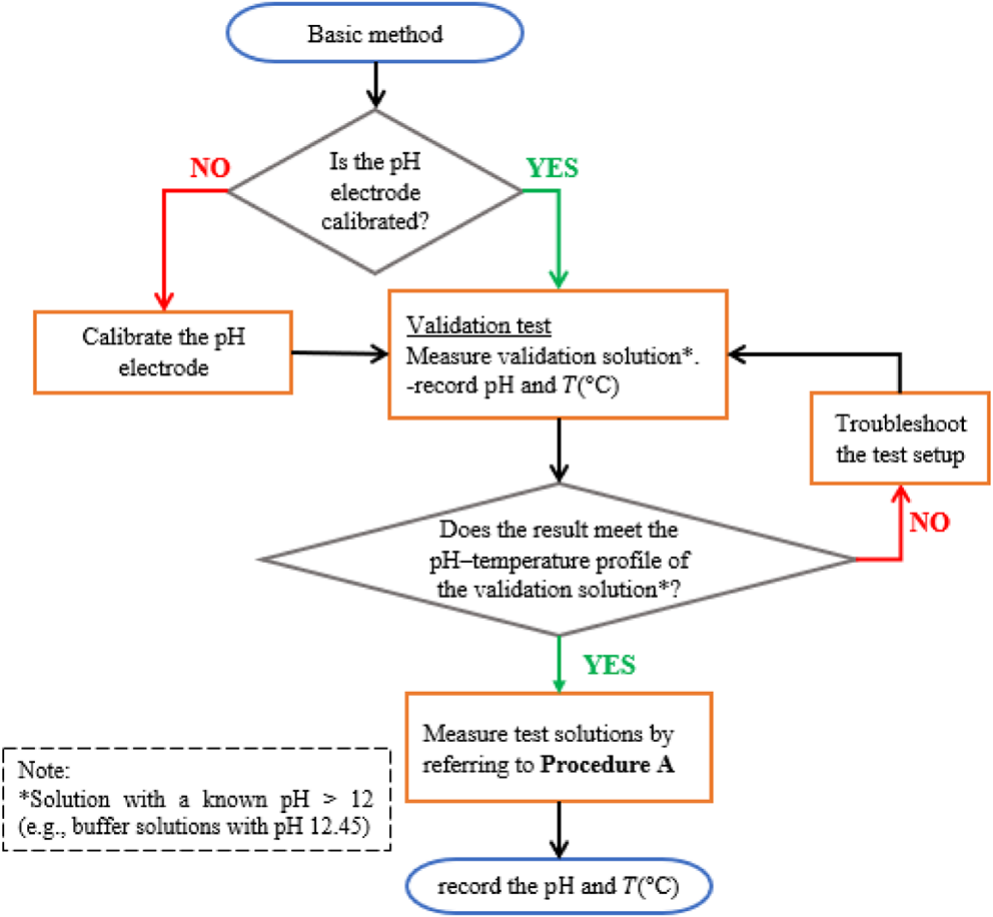
Flowchart of the proposed basic pH measurement method for cement-based materials.

**Figure 4:**
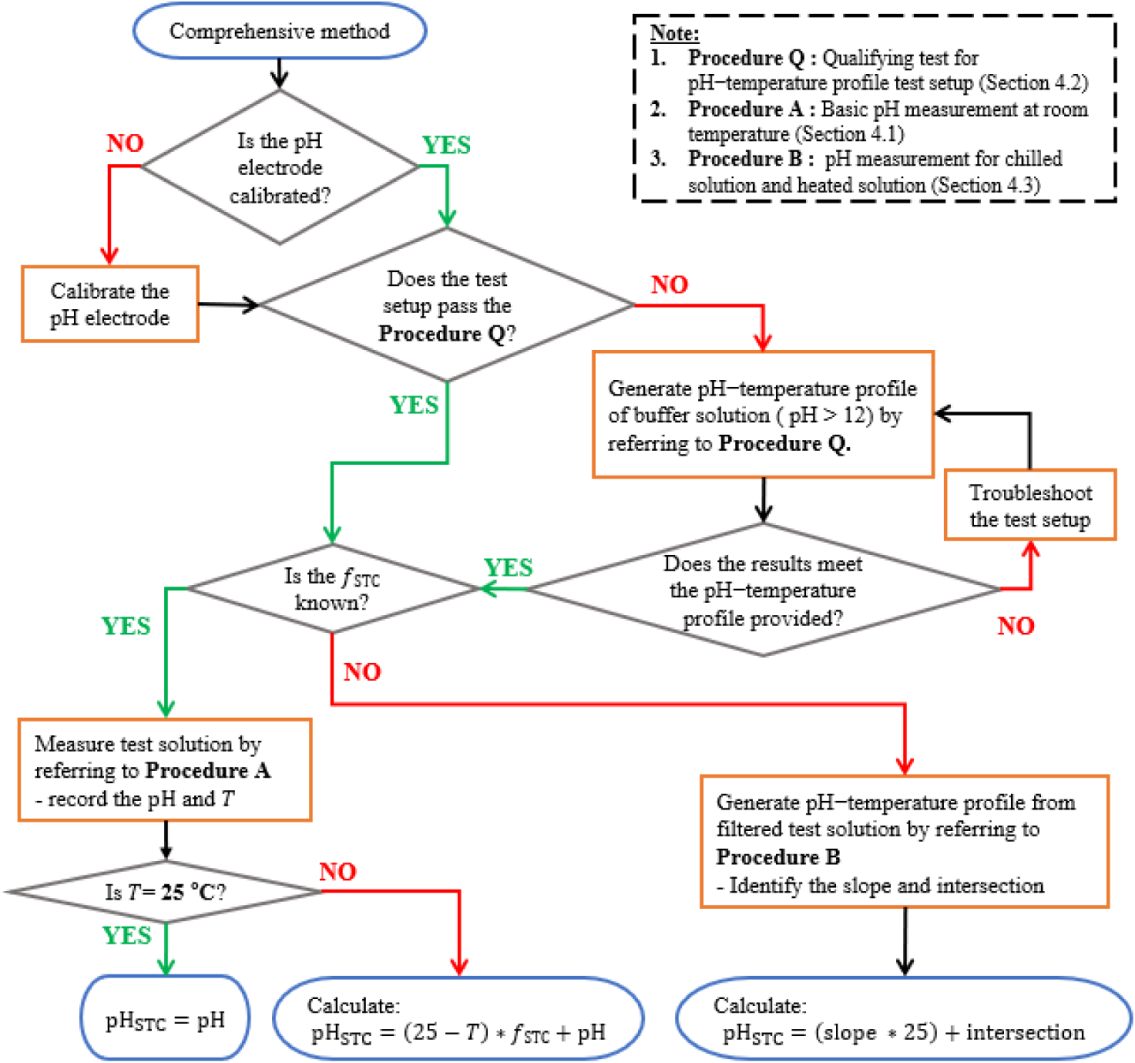
Flowchart of the proposed comprehensive pH_STC_ measurement method for cement-based materials.

As shown in Figure 3, a test is performed to validate the test setup, especially when a buffer solution with pH > 12 is not used during calibration. A calibrated pH electrode does not guarantee accuracy at high pH in measurement mode. The validation solution should be a buffer solution with pH >12 and with a known pH–temperature profile. Otherwise, it can be replaced with the filtrate of a freshly prepared saturated calcium hydroxide solution. Saturated calcium hydroxide has good buffering of pH 12.45 at 25 °C, with a pH–temperature coefficient of −0.033 pH/°C.^1^ The pH and temperature readings should meet the pH–temperature profile of the validation test solution. The advantage of using this basic procedure is that it can validate the capacity of the test setup to measure high-alkaline solutions accurately. Procedure A shows the steps suggested by the authors, referenced from the guidelines presented in Section 3.

Figure 4 shows the flowchart of the proposed comprehensive pH_STC_ measurement method for CBMs. This test requires a test setup that can generate the highly alkaline pH–temperature profile accurately (e.g., using a research-grade pH meter). For process control, when the solution temperature-compensated factor (*f*_STC_) is known, the pH-temperature profile does not need to be repeatedly generated, and pH_STC_ can be calculated directly, as shown in Figure 4.

### 4.1 Procedure A: Basic pH Measurement at Room Temperature

This procedure is designed for the preparation of 20 g of test solution by using laboratory apparatus, as shown in Figure 5. If a different volume is used, the apparatus should be adjusted accordingly. The procedure is as follows:

1 Crush the solid samples until the entire specimens can pass through sieve #30 (<600 μm).
2 Add 10 ± 0.1 g of solids to 20 ± 0.1 g of distilled water in a 60 mL screw-capped container, together with a stirrer bar (if magnetic stirring is applied). Cap the container and stir the suspension continuously for 1 min, followed by leaching for 10 min without stirring.
3 Thirty seconds before filtering the suspension, shake the suspension by hand 2–5 times, then allow sedimentation to occur before filtration via the syringe filtering method.
4 Discharge the filtrate into a 50 mL plastic tube, with at least 12.5 mL of the solution for pH measurement.
5 Measure the pH of the filtrate three times by using a calibrated pH electrode. While measuring, lightly stir the solution by using the pH electrode to equilibrate the ions in the solution.
6 The mean pH and mean temperature determine the pH of that solution temperature.

**Figure 5:**
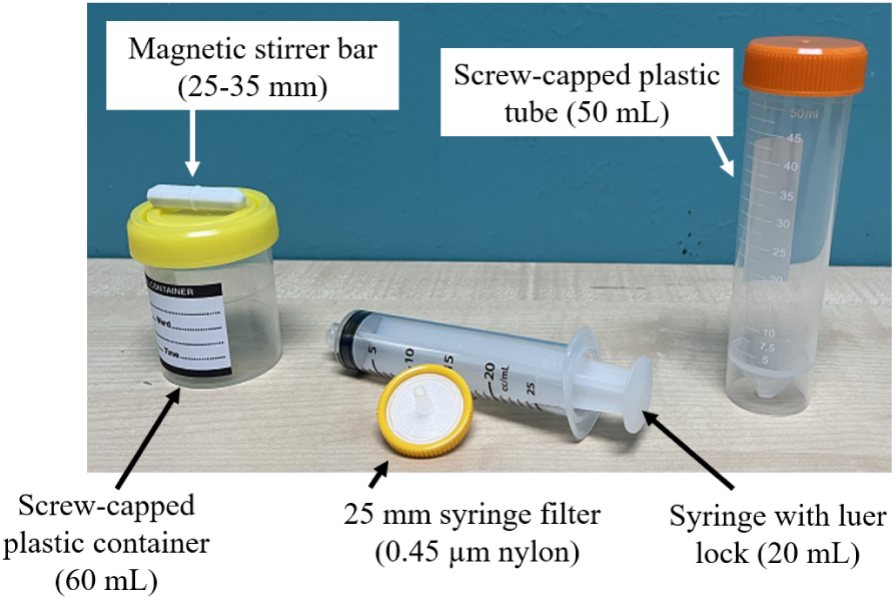
Laboratory apparatus suitable for the preparation of 20 g of test solution. (source: authors).

### 4.2 Procedure Q: Qualifying Test for pH–Temperature Profile Test Setup

The test setup must be suitable for testing to generate the pH–temperature profile for a high-pH solution. Therefore, procedure Q is introduced. The concept for this procedure is in the nature of the calibration method; the result produced from the setup should be similar to the qualified reference. The qualified reference used in this test is a technical buffer solution from the commercial market, where the pH–temperature profile of the high-pH buffer solution has been tested and released. The pH meter should be set to the “stability-accurate” data logging mode or its equivalent depending on the model of the pH meter. The details of the procedure are as follows:

1 Prepare two sets of buffer solution of pH 12.45 (or buffer solution of pH > 12 that is compatible with the pH meter used). Each set consists of 20 mL of the solution in the 50 mL tube container. Cap-seal the containers. One set is placed in an ice water bath for about 10 min to achieve a solution temperature of about 15 °C, and one set is placed in a hot water bath for about 10 min to achieve a solution temperature of about 35 °C. The temperatures of the ice and hot water baths and the soaking time are influenced by the ambient temperature and volume of water. Therefore, trials should be conducted to achieve a test solution temperature of approximately 15 and 35 °C. The selected temperature range of 10 °C above and below 25 °C covers the ambient temperature of most laboratories worldwide, and high-pH solutions exhibit high linearity within this range.
2 Remove the sample test set from the ice and hot water bath. Alternate the pH measurement between the cold and hot samples until the solution reaches a similar temperature (room temperature). Use the data logging function to record the pH and temperature of the test solution.
3 Plot the scatter graph of pH versus temperature. The linear regression should have a high coefficient of determination (*R* ^2^). Compare the slope of this trendline with the pH–temperature profile provided by the manufacturer (qualified reference) of the tested buffer solution. If the obtained slope of the linear regression deviates from the qualified reference, then proceed with troubleshooting on the test setup, including checking and conditioning the electrode (Section 2.2). Change the pH electrode if it still fails to generate this pH–temperature profile for high-pH solution. In ref 22, the test setup achieved a deviation of 3.5% with *R* ^2^ of 0.99.

### 4.3 Procedure B: pH Measurement for Chilled and Heated Solutions

Similar to what is done in procedure Q, two sets of test samples should be prepared to generate the pH–temperature profile of the test solution. The details of procedure B are as follows:

1 Crush the solid samples until the entire specimens can pass through sieve #30 (<600 μm).
2 Prepare two sets of suspension by adding 10 ± 0.1 g of solids to 20 ± 0.1 g of distilled water in a 60 mL screw-capped container, together with a stirrer bar (if magnetic stirring is applied) for each set. Cap the container and stir the suspension continuously for 1 min, followed by leaching for 10 min without stirring. One set of samples should be leached under the ice–water-bath condition, and another set should be leached under the hot-water-bath condition, similar to Step 1 in procedure Q.
3 Thirty seconds before filtering the suspension, remove the test set from the ice and hot water baths. Shake the suspension by hand 2–5 times, then allow sedimentation to occur before conducting filtration using the syringe filtering method.
4 Discharge the filtrate into a 50 mL plastic tube, with at least 12.5 mL of the solution for pH measurement.
5 The pH of the filtrate was measured by using a calibrated pH electrode for at least five times on each test set until the solution temperature gradually equilibrates with the room temperature. The data logging function was used to record the pH and temperature of the test solution. The measurement was alternated between the cold and hot samples until the solution reached a similar temperature (room temperature).
6 Record the pH and temperature for each measurement. Plot a scatter graph of pH versus temperature (pH–temperature profile) with a linear regression. If *R* ^2^ is unsatisfactory, troubleshoot the test setup and repeat the entire test. The slope of the graph is the solution temperature-compensated factor, *f*_STC_, and it should be a negative value.
7 Calculate pH_STC_ from the linear regression equation by substituting temperature with 25.

## 5 Application of the Proposed pH Test Methods on a Carbonated Mortar Cube

A test for comparing the pH of a carbonated mortar cube at different depths was designed by using the proposed pH and pH_STC_ method and with universal pH indicator strips to validate the proposed method.

### 5.1 Materials and Test Method

The mortar cubes in this study were prepared by using Portland cement and sand passing the no. 4 (4.75 mm) sieve. The mixture had a cement-to-sand (c/s) ratio of 1:6 and a water-to-cement ratio of 0.55. Given the low water content, the fresh mortar exhibited poor flowability and did not compact well in molds. After casting, the specimens were water-cured for 1 month and stored dry at room temperature until the day of testing. One of the mortar cubes was split in half by using a chisel and hammer, as shown in Figure 6(a). One-half was used for the pH test, and the other was employed to determine the carbonation depth in accordance with BS EN 14630 ^40^ for the identification of carbonated and noncarbonated zones (Figure 6(b)). The phenolphthalein indicator appears colorless at pH below 8.3 and turns violent red at pH >10. The measured carbonation depths are listed in Table 2.

**Figure 6:**
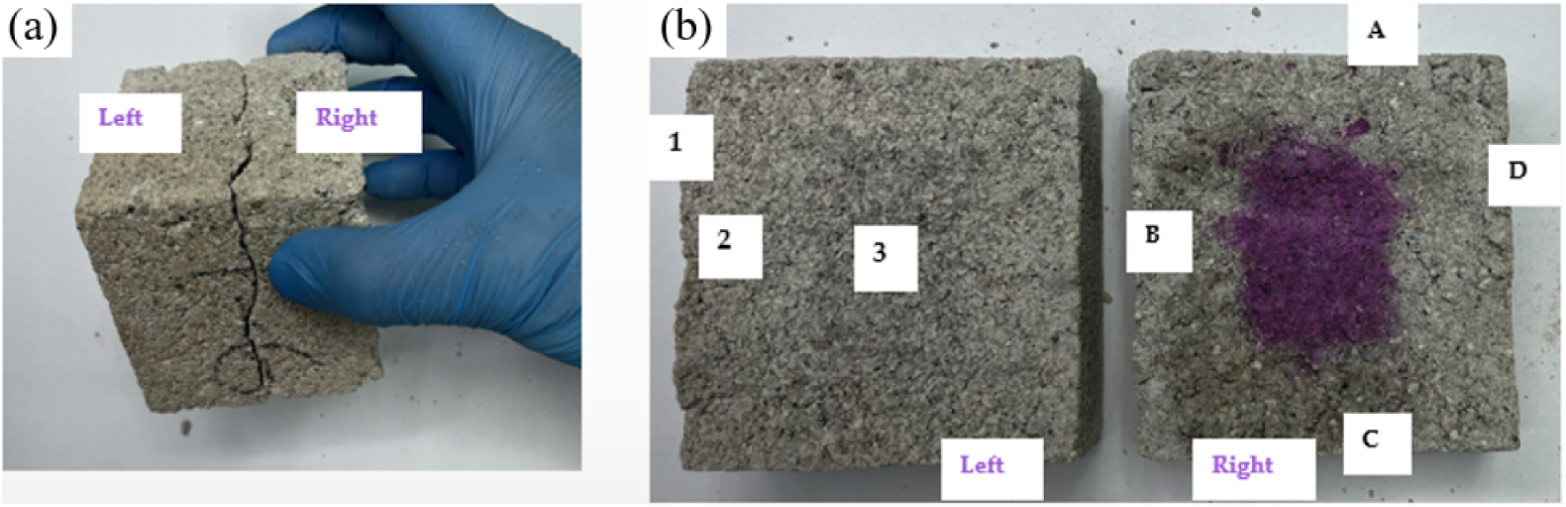
(a) Mortar cube broken into half by using a chisel and hammer. (b) Sampling zone in the left specimen and identification of the carbonated zone using phenolphthalein solution on the right specimen. (source: authors).

**Table 2:** Carbonation Depth Test Results

Location (side)	*d*_k mean_	*d*_k max_
A	17 mm	19 mm
B	20 mm	22 mm
C	19 mm	20 mm
D	21 mm	23 mm

On the basis of carbonation depth test, three types of specimens were identified and prepared for pH analysis.

1 Highly carbonated zone (<5 mm from the surface).
2 Transition Zone (<10 mm from the surface)
3 Noncarbonated zone (center).

On the left half of the cube (Figure 6(b)), which was not exposed to the phenolphthalein indicator, about 30–40 g of the solid sample was collected from each zone. Care was applied during sample separation because the noncarbonated zone contains a high concentration of Ca­(OH)_2_, which has strong buffering capacity. Therefore, the sequence of the sampling started from the outer edge of the sample. Given that the quantity of the sample available from Zone 1 on the half mortar cube was insufficient, and the sample could not be inverted without risking contamination from Ca­(OH)_2_ in the noncarbonated zone, another similar mortar cube was used to obtain a thin outer-layer sample. Sampling proceeded sequentially from Zone 1, to Zone 2, and Zone 3. Each type of solid sample was crushed to a particle size of not more than 600 μm. The pH test was performed in accordance with the method shown in Figure 4 by following the steps outlined in procedure B. The pH meter was calibrated using buffer solutions with pH 7.01, 10.01, and 12.45, and the test setup complied with the requirements of procedure Q. The pH_STC_ values were determined from the pH–temperature profile. The pH values (procedure A) were measured the on equilibrated solution handled at room temperature.

For measurement using universal pH indicator test strips, the strips were immersed directly into the unfiltered leachate until the color of the strips changed. The resulting color was then visually compared to the reference color chart provided.

### 5.2 Results and Discussion

Figures 7 and 8 show the pH–temperature profile and the results of testing using pH test strips, respectively, for the specimens collected from Zone 1, 2, and 3. The solution temperature range was approximately between 15 and 35 °C, which is sufficient for pH–temperature profile generation. The pH–temperature profile generated had high *R* ^2^ of more than 0.95.

**Figure 7:**
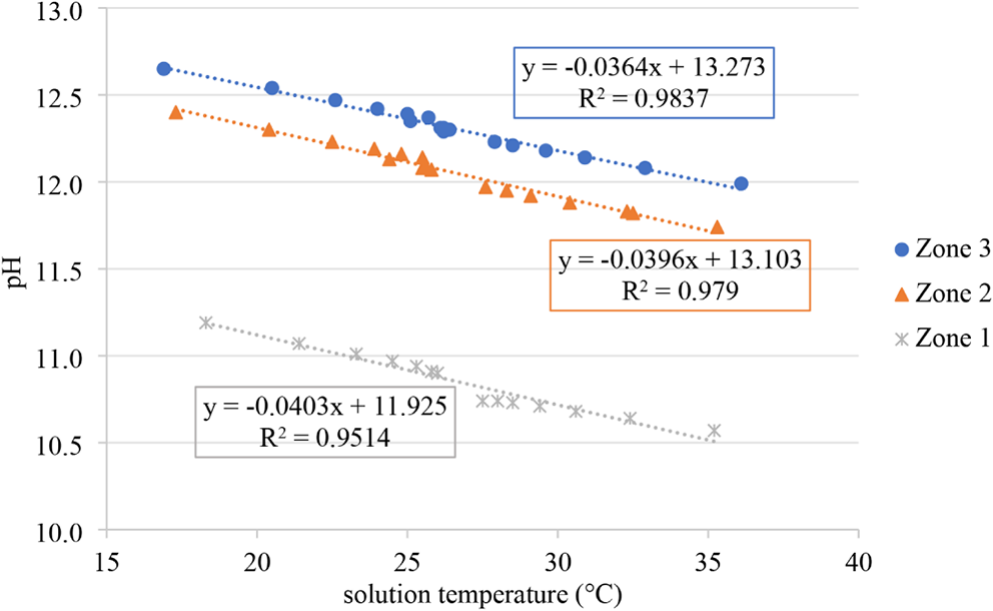
pH–temperature profile of specimens from Zone 1, 2, and 3.

**Figure 8:**
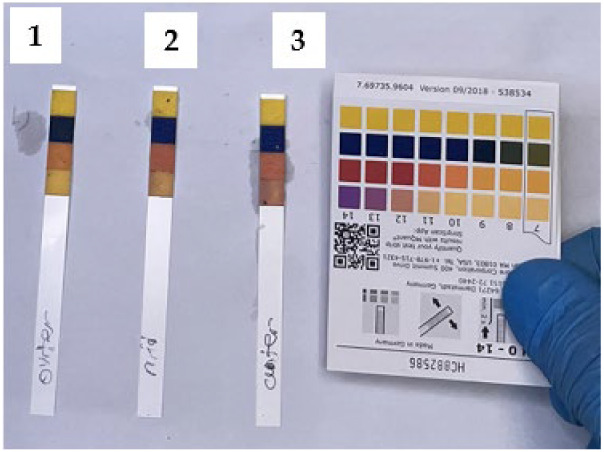
Results of pH test using universal pH indicator test strips on the three zones. (source: authors).

The results of the pH test using a pH meter and universal pH indicator test strips are summarized in Table 3. These methods showed an increasing pH trend from the outer surface to the inner core of the specimens. The developed method for determining pH_STC_ provided results that are independent of the solution temperature and suitable for direct comparison. The pH measured at ambient temperature showed that not all solutions had the same temperature, even though they were placed in the same environment. The low-pH test solutions exhibited a high *f*_STC_ value, whereas the results in ref 22 show the opposite trend. This dissimilarity may be due to differences in the chemical composition of the solutions, and further investigation is warranted.

**Table 3:** Results of pH_STC_ and pH Measured Using pH Electrode and Universal pH Indicator Test Strips at Room Temperature

Zone	*f*_STC_	*R* ^2^	pH_STC_	pH	pH (test strips)
1 (<5 mm from surface)	–0.0403	0.9514	10.92	10.90 at 26.0 °C	10
2 (<10 mm from surface)	–0.0396	0.9790	12.11	12.09 at 25.6 °C	11
3 (center – uncarbonated area)	–0.0364	0.9837	12.36	12.30 at 26.3 °C	12

## 6 Limitations of the Proposed Method

The proposed method has not been tested on alkali-activated materials (AAMs) or geopolymers that contain high levels of sodium ions. The presence of an alkaline error in the pH electrode due to sodium ions may lead to a low pH reading. Given that the sodium content in AAMs is much higher than that in Portland CBMs, this issue is particularly important and should not be overlooked. In addition, the temperature range adopted in this research was approximately between 15 and 35 °C; further study is needed to validate the proposed method for temperatures beyond this range.

Errors can occur during sampling, sample preparation, and measurement; therefore, care should be taken at each step. Examples include preventing free lime from uncarbonated concrete from contaminating the carbonated samples and always checking the performance of the pH electrode with buffer solutions of pH 12.45 or saturated calcium hydroxide solutions when measuring high-pH solutions.

## 7 Conclusions

The challenges in pH measurement of cement-based materials (CBMs) by using a pH meter stem from the high alkalinity of these materials, the fact that they do not naturally exist in aqueous form, the limited sample sizes available, and their susceptibility to carbonation. From preparing representative aqueous solutions to performing pH measurements, multiple variables must be considered; these variables include particle size and water-to-solid (w/s) ratio for leaching, leaching technique, filtration of the suspension, suitability and performance of the pH electrode, choice of buffer solutions for calibration, protection of specimens from carbonation, and solution temperature during measurement. Given the influence of these variables, a guideline was developed in this study, and two measurement methods were proposed: a basic method that reports the measured pH and temperature, and a comprehensive method that reports the pH_STC_, which represents pH standardized to 25 °C. The comprehensive method is particularly suitable for comparing high-pH samples with a pH difference of less than 1 and for pH monitoring across different seasons. Regardless of the method chosen, the performance of the pH electrode should be validated using a known high-pH reference solution after calibration (Figure 3).
